# A Novel Betaproteobacterial Agent of Gill Epitheliocystis in Seawater Farmed Atlantic Salmon (*Salmo salar*)

**DOI:** 10.1371/journal.pone.0032696

**Published:** 2012-03-12

**Authors:** Elena R. Toenshoff, Agnar Kvellestad, Susan O. Mitchell, Terje Steinum, Knut Falk, Duncan J. Colquhoun, Matthias Horn

**Affiliations:** 1 Department of Microbial Ecology, University of Vienna, Vienna, Austria; 2 Department for Basic Sciences and Aquatic Medicine, Norwegian School of Veterinary Science, Oslo, Norway; 3 Vet Aqua International, Oranmore, Ireland; 4 Department of Zoology, School of Natural Sciences, Trinity College Dublin, Dublin, Ireland; 5 Department of Laboratory Services, Norwegian Veterinary Institute, Oslo, Norway; University of California Merced, United States of America

## Abstract

Epitheliocystis, a disease characterised by cytoplasmic bacterial inclusions (cysts) in the gill and less commonly skin epithelial cells, has been reported in many marine and freshwater fish species and may be associated with mortality. Previously, molecular and ultrastructural analyses have exclusively associated members of the *Chlamydiae* with such inclusions. Here we investigated a population of farmed Atlantic salmon from the west coast of Norway displaying gill epitheliocystis. Although ‘*Candidatus* Piscichlamydia salmonis’, previously reported to be present in such cysts, was detected by PCR in most of the gill samples analysed, this bacterium was found to be a rare member of the gill microbiota, and not associated with the observed cysts as demonstrated by fluorescence *in situ* hybridization assays. The application of a broad range 16 S rRNA targeted PCR assay instead identified a novel betaproteobacterium as an abundant member of the gill microbiota. Fluorescence *in situ* hybridization demonstrated that this bacterium, tentatively classified as ‘*Candidatus* Branchiomonas cysticola’, was the cyst-forming agent in these samples. While histology and ultrastructure of ‘*Ca.* B. cysticola’ cysts revealed forms similar to the reticulate and intermediate bodies described in earlier reports from salmon in seawater, no elementary bodies typical of the chlamydial developmental cycle were observed. In conclusion, this study identified a novel agent of epitheliocystis in sea-farmed Atlantic salmon and demonstrated that these cysts can be caused by bacteria phylogenetically distinct from the *Chlamydiae*.

## Introduction

With an increasing demand for fish and overfishing of the oceans, intensive aquaculture production has increased rapidly in recent decades. In 2008, 37% of the global total production of fish, crustaceans and molluscs were obtained through aquaculture, corresponding to an economic value of over 98 billion USD (ftp://ftp.fao.org/FI/STAT/summary/YB_Overview.pdf; 8.7.2011)., Despite relatively intensive research, infectious diseases continue to represent a major challenge to aquaculture production, and much remains to be discovered relating to the aetiology and pathogenesis of infectious diseases.

The term epitheliocystis has been widely used to describe cytoplasmic membrane-bound inclusions containing Gram-negative bacteria found in gill, and less commonly, skin epithelial cells of fish. Epitheliocystis has been observed in more than 50 freshwater and marine wild and cultured fish species [1], [2], [3]. Extensive host tissue reactions and mortality due to such infections have however, only been reported in farmed fish [4], [5], [6], [7], [8], [9], [10], [11]. The presence of these cysts has also been implicated in proliferative gill inflammation (PGI) in sea-farmed Atlantic salmon, although the aetiology of PGI is not well understood [12], [13]. Several ultrastructural studies have shown the bacterial agents associated with epitheliocystis to represent a wide range of morphological forms including, in some fish species, an array of morphotypes similar to chlamydial reticulate, intermediate and elementary bodies [4], [5], [8], [14], [15], [16], [17], [18].

Using molecular, cultivation-independent methods, epitheliocystis in farmed salmonids has been associated recently with several novel *Chlamydiae*. These include ‘*Candidatus* Piscichlamydia salmonis’ in Atlantic salmon and Arctic char in sea- [12], [19] and freshwater [20] respectively, *Neochlamydia*-like bacteria found in Arctic char in freshwater [21], and ‘*Candidatus* Clavochlamydia salmonicola’ found in Atlantic salmon in freshwater [17], [22]. Such molecular studies have contributed to an increased understanding of the genetic diversity and wide host range of *Chlamydiae* [18]. However, the ability to cause epitheliocystis might not be restricted to the *Chlamydiae*. During a recent study of salmon populations from seawater displaying PGI, a significant discrepancy between the number of histologically observed cysts and the occurrence of ‘*Ca.* P. salmonis’ estimated by quantitative PCR was registered [12]. As samples were also negative for ‘*Ca.* Clavochlamydia salmonicola’, it was suggested that other as yet unidentified bacteria were responsible for many of the observed inclusions in these fish. In the present study we identified a novel betaproteobacterium in gill cysts of seawater farmed Atlantic salmon displaying PGI. The name ‘*Candidatus* Branchiomonas cysticola’ is proposed for this novel cyst-forming agent.

## Materials and Methods

### Tissue sampling

All samples were taken by a qualified veterinarian as part of a disease diagnostic investigation. Sampled fish were euthanized humanely prior to sampling. No permit is required for such diagnostic work in Norway. Gill samples were taken from a population of seawater farmed Atlantic salmon, affected by PGI, in south-western Norway, during the autumn of 2007. Tissues from the ventral parts of the second and third gills were directly fixed for histology and fluorescence *in situ* hybridisation (FISH). For transmission electron microscopy and DNA isolation, tissues were freshly frozen, or collected in RNAlater (Ambion) and stored at −80°C.

### Histological examination

Dissected gills were fixed in 10% neutral phosphate-buffered formalin for three days at room temperature and subsequently embedded in paraffin using a standard protocol, sectioned and stained with haematoxylin and eosin (HE) according to standard histological techniques [23]. Single sections including gill filaments and lamellae from each fish were examined by light microscopy. Selected sections were also Gram-stained. Each fish was examined with respect to pathological changes to investigate the severity of PGI and to count the number of cysts within gill tissues using a grading system [slight/low numbers (1), moderate/moderate numbers (2), severe/large numbers (3)].

### Transmission electron microscopy

Gills from three fish displaying epitheliocystis were examined. Tissues were fixed in 3% glutaraldehyde in 0.1 M cacodylate buffer (pH 7.4) and stored at 4°C, washed in 0.1 M sodium cacodylate buffer at pH 7.4, postfixed in a mixture of 2% (w/v) osmium tetroxide and 1.5% (w/v) potassium ferri hexacyanide in cacodylate buffer, washed, passed through a graded ethanol series and propylene oxide, and embedded in Lx-112 medium (Ladd Research Industries, Inc., Burlington, Vermont, UK). Ultra-thin sections were contrasted with uranyl acetate and lead citrate, and examined with a Philips EM 208 S electron microscope at 60 kV.

### Enrichment of gill-associated bacteria and DNA purification

Gills were homogenised and suspended in buffer A (35 mM Tris-HCl, 25 mM KCl, 10 mM MgCl_2_, 250 mM sucrose, pH 7.5) [24] containing 2 mg/ml Pronase E (Sigma), incubated for 35 min at 37°C and subsequently centrifuged for 10 min at 6,000 rpm at 4°C. The pellet was resuspended in buffer A containing 250 mM ETDA and again homogenized with a Dounce tissue grinder (Wheaton) and filtered through a 5 µm syringe filter. The suspension was centrifuged as before, the pellet washed twice with buffer A and then resuspended in buffer A containing 10 units DNase I. The sample was incubated for 1 h at 4°C followed by DNase inactivation with 50 mM EDTA. The suspension was centrifuged, the pellet washed with buffer A containing 250 mM EDTA and resuspended in TE buffer (10 mM Tris-HCl, 1 mM EDTA, pH 7.5). DNA was purified using a sodium dodecyl sulphate (SDS)-based method including 1% hexadecylmethylammonium bromide (CTAB) and 200 µg/ml Proteinase K (Roche Applied Science) in the extraction buffer [25]. DNA was stored at −20°C until further use.

### PCR, cloning, RFLP, and sequencing

Partial 16 S rRNA gene sequences were amplified by PCR and sequenced as described in Table S1. Novel primers specific for ‘*Ca.* P. salmonis’ were designed using the probedesign/probematch tool implemented in the ARB software package [26](Table S1). PCR reactions consisted of 2 µl template DNA, 1 unit of Taq DNA Polymerase (Fermentas), 10× Taq buffer with KCl and 2 mM MgCl_2_, 0.2 mM of each deoxynucleotide and 50 pmol of each primer in a total volume of 50 µl. Negative (no DNA added) and positive controls were included in all PCR reactions. The presence and size of amplicons were checked by gel electrophoresis and ethidium bromide or Syber Green staining. PCR products were purified using a PCR purification kit (Qiagen) and either directly sequenced or cloned using the TOPO TA Cloning® kit (Invitrogen Life Technologies) according to the manufacturer's instructions. Fifteen to 30 clones were screened and analyzed by restriction length polymorphism (RFLP) analysis using the enzyme *MspI* (Fermentas). PCR products or clones were sequenced using the BigDye Terminator kit v3.1 and an ABI 3130 XL Genetic Analyzer.

### Quantitative PCR (qPCR)

Approximately 17.5 mg of gill soft tissues (preserved in RNAlater) were homogenized with a Roche MagNA lyser (Roche Ltd., Basel, Switzerland). DNA was extracted from half the homogenate volume using the Roche High Pure PCR Template Preparation kit (Roche) according to the manufacturer's instructions. The suitability of the purified DNA for qPCR was verified with an elongation factor alpha 1 PCR assay and the samples examined for the presence of ‘*Ca*. P. salmonis’ as described previously [12].

### Fluorescence *in situ* hybridization (FISH)

Fluorescence *in situ* hybridization was performed either on sections of gill tissues prepared for histological analysis, or on fresh, frozen gill tissues fixed separately. For the latter, gill samples were squashed and fixed in 4% paraformaldehyde at 4°C for 1 h and subsequently washed in phosphate buffered saline. An aliquot of this suspension was then dropped on a glass slide, dried at 46°C and used for FISH. Standard hybridization conditions, hybridization and washing buffers were used [27]. Oligonucleotide probes used are given in Table S1. New probes were designed using the probedesign/probematch tools of the ARB software package [26] and deposited at probeBase [28]. Probe NONEUB was used as negative control. Optimal hybridization conditions for the newly designed probes were determined in a series of hybridization experiments with increasing formamide concentrations in the hybridization buffer. Slides were examined using a confocal laser scanning microscope (LSM 510 Meta, Carl Zeiss) equipped with two helium-neon-lasers (543 nm and 633 nm) and an argon laser (458–514 nm). Standard software delivered with the instrument (version 3.2) was used for image acquisition.

### Clone-FISH

Clone-FISH was performed to evaluate the ‘*Ca.* P. salmonis’ specific oligonucleotide probe (Table S1). The vector pCR®2.1-TOPO® (Invitrogen Life Technologies) containing the amplified ‘*Ca.* P. salmonis’ 16 S rRNA gene fragment was transformed into *E. coli* JM109 (DE3) cells and the insert was *in vivo* transcribed to generate target-rRNA as described [29]. *E.coli* cells were fixed with 4% paraformaldehyde for 30 min at RT and used for FISH following the standard protocol [27].

### Phylogenetic sequence analysis

The ARB program package [26] was used for phylogenetic analysis. An in-house 16 S rRNA sequence database updated using blastn homology search for the newly obtained sequences with sequences deposited in the GenBank database provided by the NCBI (National Centre for Biotechnology Information) was used [30]. Trees were calculated using TREEPUZZLE with the HKY evolutionary model of substitution and maximum parsimony (1000 replicates) implemented in ARB [31], [32]. PhyML trees (HKY85, 1000 replicates) were calculated using PHYML 3.0 provided by the Mobyle portal (http://mobyle.pasteur.fr/cgi-bin/portal.py) [33]. The program MEGA [34] was used for the distance method Neighbor-Joining (Jukes-Cantor correction, 1000 bootstrap replicates).

### Nucleotide sequence accession numbers

The obtained 16 S rRNA gene sequences of ‘*Ca.* B. cysticola’ and ‘*Ca.* P. salmonis’ were deposited at GenBank/EMBL/DDBJ under the accession numbers JN968376 and JQ065095/JQ065096 respectively.

## Results

### Histology and electron microscopy

**Figure 1:**
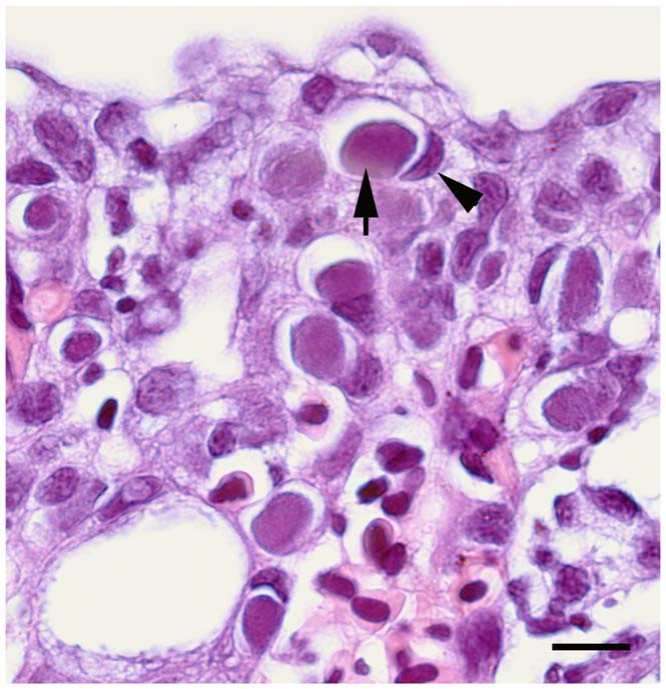
Epitheliocystis in gills of Norwegian seawater farmed Atlantic salmon (*Salmo salar* L.). Haematoxylin and eosin stained sections from formalin-fixed and paraffin embedded gill tissues. The cysts (arrow) appeared in epithelial cells as regular rounded to oval, granular, basophilic (blue) and well-circumscribed cytoplasmic inclusions occupying most of the cell volume. The host cell nuclei were flattened and displaced (arrowhead). Scale bar represents 10 µm.

A population of seawater farmed Atlantic salmon showing signs of respiratory distress was investigated. Of the 15 sampled fish, 14 displayed pathological changes consistent with PGI, briefly; circulatory disturbance, inflammation, epithelial cell-death and hyperplasia [13], while cysts containing Gram-negative bacteria were observed in 12 (Figure 1).

**Figure 2:**
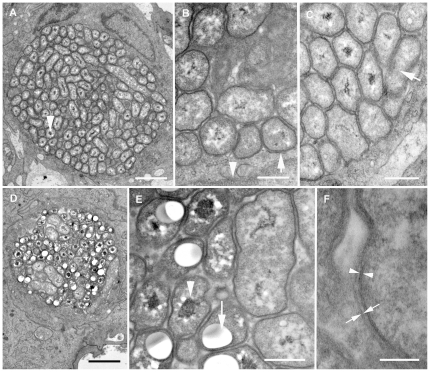
Cyst and bacterial ultrastructure. Ultra-thin sections of gill tissue examined by transmission electron microscopy. The micrograph depicts cyst morphology in fish gill tissues negative for the presence of ‘*Ca.* P. salmonis’ by qPCR. (**A**) The cysts were densely packed with polymorphic bacteria of round, coccoid or short to long rod-shaped morphologies, with or without nucleoids (electron dense material in the centre of the bacterium, arrowhead). Scale bar represents 2 µm. (**B**) Cysts were limited by a membrane formed by the host cell (arrow). Plasma membrane of the host cell (arrowhead). Electron dense nucleoids were observed in many bacterial cells. Scale bar represents 500 nm. (**C**) Nucleoids were apparently absent in a low number of bacteria, resembling morphotypes described as chlamydial reticulate bodies in previous studies (arrow). Scale bar represents 500 nm. (**D**) Other cysts contained more bacterial cells with distinct nucleoids resembling morphotypes previously described as chlamydial intermediate bodies. Scale bar represents 2 µm. (**E**) A high number of the cyst associated bacteria contained large nucleoids (arrowhead) and vesicles (arrow), which may have artefactually expanded during tissue processing, compressing the surrounding structures. Scale bar represents 500 mn. (**F**) All bacteria were limited by a double membrane of which the inner (arrowhead) was clearly trilaminar while the outer (arrows) was less distinct. Scale bar represents 100 nm.

Electron microscopy was performed on gill tissues from three fish, displaying cysts and low loads of ‘*Ca*. P. salmonis’. The cysts appeared as large membrane-bound cytoplasmic inclusions containing pleomorphic bacterial cells with a Gram-negative type cell wall (Figure 2A–F). Rounded to elongated forms, approximately 0.2–0.4 (diameter)×≤2 µm (length) were observed, with a number of the rounded type containing small vesicles possibly consisting of storage compounds. Most of the observed morphotypes resembled the intermediate- while few resembled the reticulate- developmental forms of *Chlamydiae* described in Atlantic salmon [16], [19] and warm-blooded animals [35]. Elementary body-like morphotypes were not observed.

### ‘Ca. P. salmonis’ not present in cysts

Samples from all 15 fish were examined by qPCR with a ‘Ca. P. salmonis’-specific assay [12]. Twelve fish were positive and gave Ct values between 26.9 and 35.5 (median 31.6) suggesting a typical load similar to the estimated ≤1360 cells mg^−1^ soft tissue previously reported [12]. Only one sample in the present study was considered as having a high ‘Ca. P. salmonis’ load (Ct value 26.9) and notably no cysts could be histologically observed in this fish. Although this study has examined too few samples to conclude on the association between ‘Ca. P. salmonis’ and cysts, no or moderate loads were detected by qPCR in gills of eight of the 12 fish displaying both severe PGI and large numbers of cysts. Thus our results support the previously reported lack of association between ‘Ca. P. salmonis’ load and cyst number [12].

This suggests the presence of another agent of epitheliocystis in these fish. Yet, ‘*Ca.* P. salmonis’ was clearly present in most of the samples. To further investigate, we analysed a fish with severe PGI, large numbers of cysts and a high Ct value (35.5), reflecting a low load of ‘*Ca*. P. salmonis’, using a *Chlamydiae*-specific PCR assay combining a general chlamydial- and a universal 16 S rRNA gene-targeted primers (SigF2/Univ1390R; Table S1). Cloning of the obtained PCR product and RFLP analysis of 30 clones revealed 14 different RFLP patterns. One representative clone of each RFLP type was sequenced. We recovered one 16 S rRNA gene sequence (represented by three of 30 clones analyzed) identical to ‘*Ca.* P. salmonis’ from a farmed Atlantic salmon population from Norway (99.9–100% sequence similarity) [19]. The remaining sequences were related to *Planctomycetes* commonly found in sea water; no other sequence related to known members of the *Chlamydiae* was found. Additional analysis of four other fish using *Chlamydiae*-specific (primers SigF2/SigR2) and ‘*Ca.* P. salmonis’ specific (primers Pisci211F/Pisci1353R, Table S1) PCR assays confirmed these findings. Identical ‘*Ca.* P. salmonis’ 16 S rRNA genes, but none similar to other *Chlamydiae*, were detected in all specimens. Taken together, the available evidence suggests that ‘*Ca.* P. salmonis’ was a rare member of the microbial community associated with the gills of the studied fish, and that no other bacteria related to known *Chlamydiae* were present.

**Figure 3:**
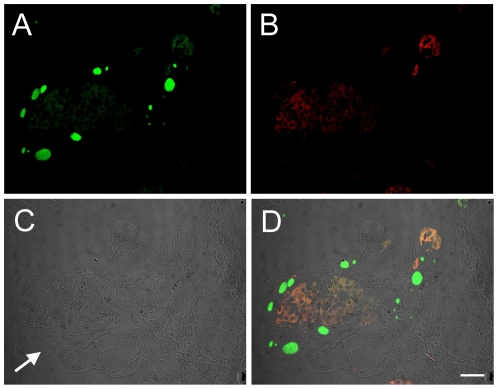
Absence of ‘*Ca.* Piscichlamydia salmonis’ in cysts. Fluorescence *in situ* hybridization of sections of gill tissues using (**A**) a general bacterial probe mix labelled with Fluos (green), and (**B**) the ‘*Candidatus* P. salmonis’ specific probe Psc-523 labelled with Cy3 (red) simultaneously. The faint red signal represents autofluorescence of the gill tissues. (**C**) Digital interference contrast image showing cysts (arrow) in the epithelial cells. (**D**) The overlay of all three images demonstrates the presence of bacteria in the cysts, which hybridize with the bacterial probe mix but not with the ‘*Ca.* Piscichlaymdia salmonis’ specific probe and thus appear green. Additional staining with the DNA stain 4′,6-diamidino-2-phenylindole (DAPI) confirmed that all bacteria present in the gill tissues hybridized with the general bacterial probe mix. Scale bar represents 20 µm.

To verify our PCR-based analysis we performed fluorescence *in situ* hybridization (FISH). For the specific detection of ‘*Ca.* P. salmonis’ we designed a novel oligonucleotide probe (Psc-523) and used the Clone-FISH method [29] to show that this probe successfully detects ‘*Ca.* P. salmonis’ 16 S rRNA and to determine its optimal hybridization conditions (Table S1, Figure S1). We then analysed three of the fish for which we had qPCR and PCR evidence for the presence of ‘*Ca.* P. salmonis’. Simultaneous application of the ‘*Ca.* P. salmonis’ specific probe and a general bacterial probe-mix targeting most known *Bacteria* readily visualized cysts containing bacteria, but did not result in hybridisation of the ‘*Ca.* P. salmonis’ specific probe and bacterial cells within inclusions (Figure 3). Only occasionally, faint signals with the ‘*Ca.* P. salmonis’ specific probe were apparent, diffusely distributed throughout the tissues, which might represent single bacterial cells or small cell clusters of ’*Ca.* P. salmonis’ external to cysts. This confirmed our PCR-based results and demonstrated clearly that the cysts in these gill samples contained bacteria other than ‘*Ca.* P. salmonis’.

### Novel betaproteobacterium associated with epitheliocystis

To identify the bacteria associated with epitheliocystis in these fish, we used a broad PCR assay targeting the 16 S rRNA genes of most *Bacteria* (primers 616V/1492R; Table S1). RFLP analysis of the cloned PCR products from one fish revealed three different patterns. The clone representing the most abundant RFLP pattern (comprising 7 of 15 clones) showed only low sequence similarity to known *Betaproteobacteria* (around 88%), but was nearly identical (99.2%) to a partial 16 S rRNA gene sequence identified by denaturing gradient gel electrophoresis (DGGE) during a recent survey of gill-associated microbiota in Atlantic salmon [36].

**Figure 4:**
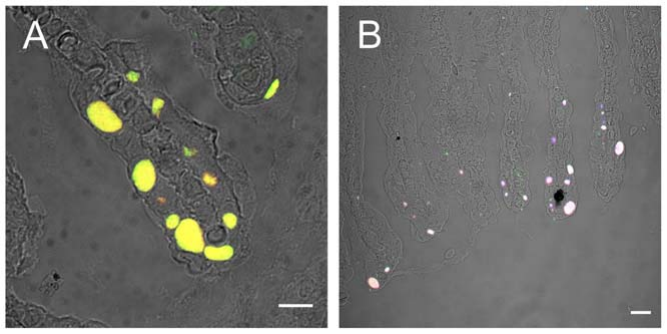
*In situ* identification and localization of ‘*Ca.* Branchiomonas cysticola’ within cysts. Overlays of digital interference contrast (DIC) and fluorescence images of gill tissue sections are shown. (**A**) Hybridization with the ‘*Ca.* B. cysticola’ specific probe BraCy-129 labelled with Cy3 (red) in combination with a bacterial probe mix targeting most *Bacteria* labelled in Fluos (green). The combined fluorescence signals of both probes appear yellow. Scale bar represents 10 µm. (**B**) Hybridization with the ‘*Ca.* B. cysticola’ specific probe BraCy-129 labelled in Cy3 (red), the bacterial probe mix labelled in Cy5 (blue), and probe BTWO23A targeting a subset of the *Betaproteobacteria* labelled in Fluos (green). The combined fluorescence signals of all three probes appear white. Scale bar represents 20 µm.

To test whether this abundant phylotype was associated with cysts in the investigated fish population, we developed an oligonucleotide probe specific for this phylotype and used FISH on both sections and gill tissue squashes. The bacteria located within cysts could be readily visualized with the oligonucleotide probe BraCy-129. The simultaneous application of a bacterial probe mix and a probe targeting many *Betaproteobacteria* confirmed the absence of ‘*Ca.* P. salmonis’ and demonstrated that the novel betaproteobacterial phylotype is the only cyst-associated microbe in these samples (Figure 4).

**Figure 5:**
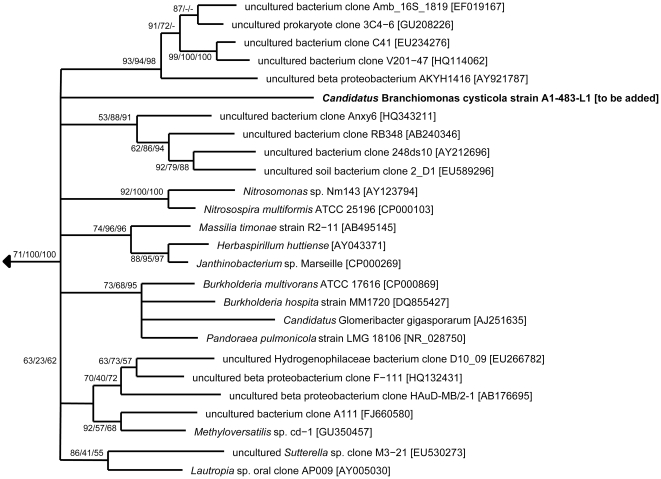
Phylogenetic relationship of ‘*Ca.* Branchiomonas cysticola’ with the *Betaproteobacteria*. A 16 S rRNA-based TREEPUZZLE tree is shown. The branching order near the root of the tree varies between different treeing methods and can thus not be reliably resolved. TREEPUZZLE support and bootstrap values for maximum parsimony, maximum likelihood and Neighbor-Joining (1000 resamplings) are indicated at the inner nodes. GenBank accession numbers are given in the square brackets. Bar, 10% estimated evolutionary distance.

Phylogenetic analysis showed that the abundant phylotype established a novel, deep branching lineage within the *Betaproteobacteria* (Figure 5). Depending on the phylogenetic analysis method used, the novel lineage clusters together with other bacteria found in diverse environments, albeit with low bootstrap values, which does not allow reliable determination of its' position within the *Betaproteobacteria*. The low degree of phylogenetic relationship of the novel gill-associated bacterium to other *Betaproteobacteria* (less than 95% 16 S rRNA sequence similarity) justifies its' classification into a new genus. According to the recommendations of Murray and Stackebrandt [37] we propose, therefore, the provisional name ‘*Candidatus* Branchiomonas cysticola’, for the novel, epitheliocystis-associated bacteria identified in this study. The proposed genus name is the combining form of gr. noun branchia (meaning gills) with gr. noun monas (meaning a unit, monad) while the species name is derived from new lat. cystis (meaning membranous sac, pouch (from gr. Kystis)) and lat. verb colere/lat. noun incola (meaning to inhabit/inhabitant or dweller).

## Discussion

Until the present study, epitheliocystis in fish gills has generally been assumed to be caused by members of the *Chlamydiae*. This assumption has been based on ultrastructural studies corroborated more recently by molecular methodology. The identification of ‘*Ca.* B. cysticola’ in this study represents the first demonstration of epitheliocystis-associated bacteria related to the *Betaproteobacteria* in any marine or freshwater fish species. This suggests that epitheliocystis is a condition that can be caused by different, evolutionary distinct bacteria.

The overall cyst morphology and the very pleomorphic bacterial cell morphotypes observed in the present study are highly similar to the intermediate and reticulate bodies reported previously [16], [19]. The electron-dense elementary bodies reported by Nylund and co-workers [16], which are not documented outside the *Chlamydiae*, were not observed. Interestingly, the intermediate body-like morphotypes are also very similar to those observed in some beta-proteobacteria causing intracellular respiratory infections in mammals [38]. Thus, while histological and ultrastructural similarities exist, the overall cell morphologies observed in the present study do not appear to be consistent with a chlamydial life cycle.

Although fluorescence *in situ* hybridization unambiguously identified ‘*Ca.* B. cysticola’ as the cyst-forming agent in the fish analysed in the present study (Figure 4), we also detected ‘*Ca.* P. salmonis’ (but no other *Chlamydiae*) by PCR. Fluorescence *in situ* hybridization demonstrated that ‘*Ca.* P. salmonis’ is in fact a rare member of the gill-associated microbiota and not responsible for the cysts in these samples (Figure 3). *Chlamydiae* have been detected and sequenced in association with epitheliocystis in a number of fish species [17], [19], [20], [21], [22], [39], [40]. While *in situ* evidence based on riboprobing (using polynucleotide probes) has been presented in some cases [17], [19], [20], [21] the link between retrieved DNA sequences and the bacterial cells within the respective cysts has not always been conclusively confirmed [22], [39], [40]. The possibility exists therefore that some of the chlamydial sequences detected by PCR and associated with epitheliocystis may in fact represent organisms external to the cysts.

In conclusion, we have identified a novel agent of epitheliocystis in sea-farmed Atlantic salmon for which we propose the name ‘*Candidatus* Branchiomonas cysticola’. The diversity in bacteria now known to be associated with epitheliocystis in seawater farmed Atlantic salmon probably explains the lack of association between ‘Ca. P. salmonis’ and observed cyst number in a previous study [12]. Future analysis should thus ideally not rely on PCR-based detection methods alone, but should include evaluation of PCR results by an *in situ* technique such as FISH. Interestingly there is molecular evidence that ‘*Ca.* B. cysticola’ is also a member of the normal gill microbiota of apparently healthy sea farmed Atlantic salmon [36]. It remains to be determined how widespread these bacteria are, and what their contribution is to the aetiology of epitheliocystis and PGI.

## Supporting Information

Figure S1 **Evaluation of the newly designed‚** ***Candidatus*** **Piscichlamydia salmonis' specific probe Psc-523 using Clone-FISH.**
(PPTX)

Table S1 **PCR primers and fluorescently labelled probes used in this study.**
(DOCX)
