# Supplementary material for: A Novel Betaproteobacterial Agent of Gill Epitheliocystis in Seawater Farmed Atlantic Salmon (Salmo salar)
Source: PLoS One. 2012 Mar 12;7(3):e32696. doi: 10.1371/journal.pone.0032696 (PMC3299688; doi:10.1371/journal.pone.0032696)
Supplement: Figure S1 — Evaluation of the newly designed‚ Candidatus Piscichlamydia salmonis' specific probe Psc-523 using Clone-FISH. (PPTX) [file pone.0032696.s001.pptx]

## Slide 1
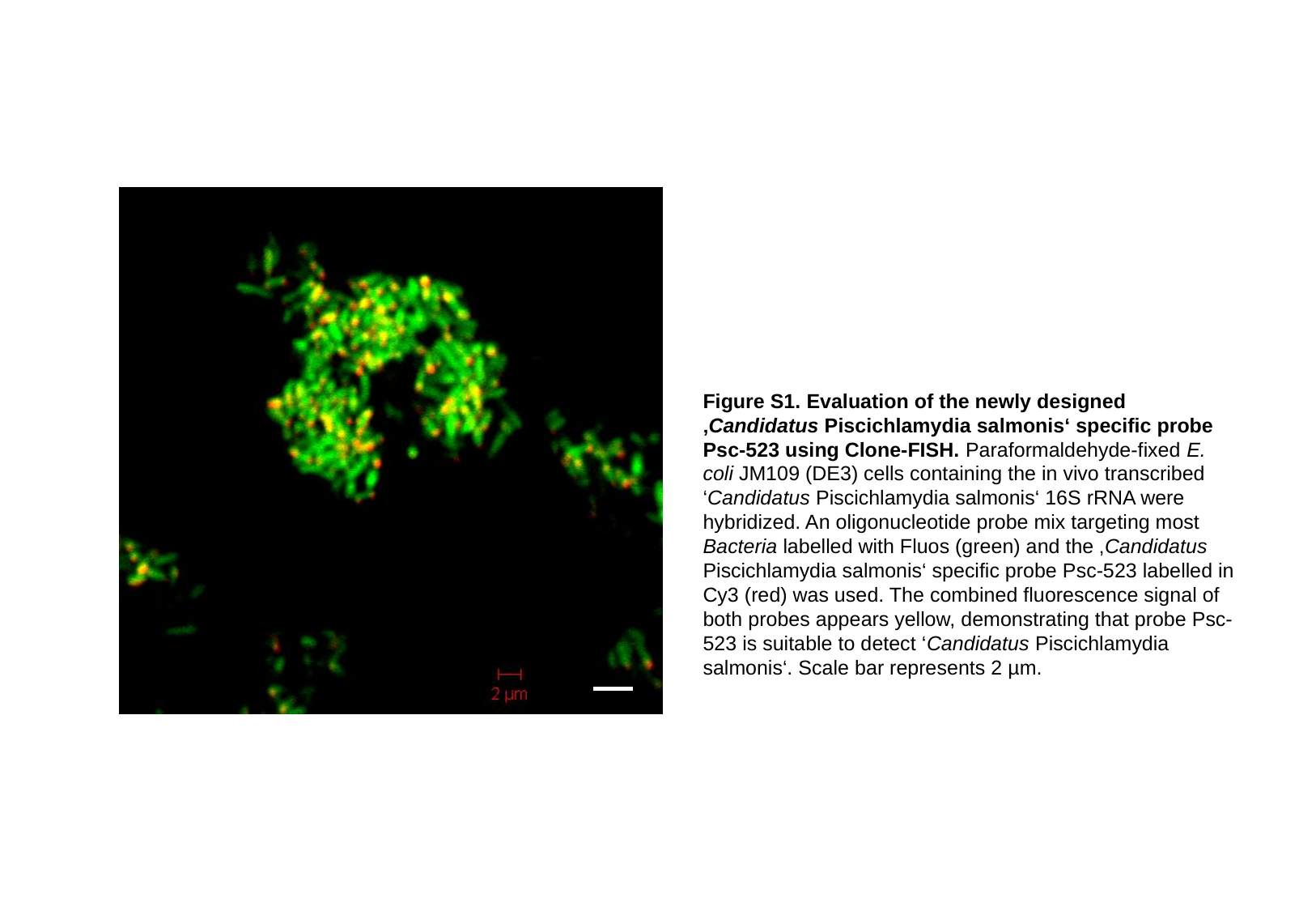

Figure S1. Evaluation of the newly designed ‚Candidatus Piscichlamydia salmonis‘ specific probe Psc-523 using Clone-FISH. Paraformaldehyde-fixed E. coli JM109 (DE3) cells containing the in vivo transcribed ‘Candidatus Piscichlamydia salmonis‘ 16S rRNA were hybridized. An oligonucleotide probe mix targeting most Bacteria labelled with Fluos (green) and the ‚Candidatus Piscichlamydia salmonis‘ specific probe Psc-523 labelled in Cy3 (red) was used. The combined fluorescence signal of both probes appears yellow, demonstrating that probe Psc-523 is suitable to detect ‘Candidatus Piscichlamydia salmonis‘. Scale bar represents 2 µm.
