# Supplementary material for: A Novel Betaproteobacterial Agent of Gill Epitheliocystis in Seawater Farmed Atlantic Salmon (Salmo salar)
Source: PLoS One. 2012 Mar 12;7(3):e32696. doi: 10.1371/journal.pone.0032696 (PMC3299688; doi:10.1371/journal.pone.0032696)
Supplement: Table S1 — PCR primers and fluorescently labelled probes used in this study. (DOCX) [file pone.0032696.s002.docx]

**Table S1. PCR primers and fluorescently labelled probes used in this study**.

| **Short name** | **Sequence (5´-3´)** | **Specificity** | **Target site**^b^ | **Annealing temperature/ Formamide concentration** | **Reference** |
| --- | --- | --- | --- | --- | --- |
| **PCR primer** | | | | | |
| **616V** | AGA GTT TGA TYM TGG CTC | 16S rRNA gene, most *Bacteria* | 8-25 | 52°C | [1] |
| **1492R** | GGY TAC CTT GTT ACG ACT T | 16S rRNA gene, most *Bacteria* and *Archaea* | 1492-1510 |  | [2] |
| **Univ1390R** | GAC GGG CGG TGT GTA CAA | 16S rRNA gene, most *Bacteria* | 1391-1407 | 56°C | [3] |
| **SigF2** | CRG CGT GGA TGA GGC AT | 16S rRNA gene, *Chlamydiales* | 40-56 | 56-60°C | [4] |
| **SigR2** | TCA GTC CCA RTG TTG GC | 16S rRNA gene, *Chlamydiales* | 309-325 | 60°C | [4] |
| **Pisci211F** | GAG CCT TGT GGT TTG AGA GC | 16S rRNA gene, ‘*Candidatus* Piscichlamydia salmonis’ | 211-230 | 70°C | This study |
| **Pisci1363R** | GAA CGT ATT CAC GGC GCT AT | 16S rRNA gene, mainly ‘*Candidatus* Piscichlamydia salmonis’ | 1363-1382 |  | This study |
| **Oligonucleotide probes** | | | | | |
| **EUB338-I** ^a^ | GCT GCC TCC CGT AGG AGT | 16S rRNA, most *Bacteria* | 338-355 | 10-50% | [5] |
| **EUB338-II** ^a^ | GCA GCC ACC CGT AGG TGT | 16S rRNA, bacteria not covered by probe EUB338-I, e.g. many *Planctomycetes* | 338-355 | 10-60% | [6] |
| **EUB338-III** ^a^ | GCT GCC ACC CGT AGG TGT | 16S rRNA, bacteria not covered by probe EUB338-I, e.g. many *Verrucomicrobia* | 338-355 | 10-60% | [6] |
| **Psc-523** | CCC ACG TAT TAC CGC AGC | 16S rRNA, ‘*Candidatus* Piscichlamydia salmonis’ | 524-541 | 35% | This study |
| **BraCy-129** | CCC ACC ACT AGA CAC GTT | 16S rRNA, ‘*Candidatus* Branchiomonas cysticola’ | 129-146 | 35% | This study |
| **BTWO23A** | GAA TTC CAC CCC CCT CT | 16S rRNA, many *Betaproteobacteria* | 663-679 | 35% | [7] |
| **BONE23A** (competitor for BTWO23A) | GAA TTC CAT CCC CCT CT | 16S rRNA, beta1-group of *Betaproteobacteria* | 663-679 | 35% | [7] |
| **NONEUB** | ACT CCT ACG GGA GGC AGC | Control probe complementary to EUB338-I | 338-355 | 10-60% | [8] |

^a^ EUB338-I, EUB338-II, and EUB338-III were applied simultaneously to target most *Bacteria*.

^b^ Target site according to *E. coli* 16S rRNA gene numbering.

**REFERENCES**

1. Juretschko S, Timmermann G, Schmid M, Schleifer KH, Pommerening-Roser A, et al. (1998) Combined molecular and conventional analyses of nitrifying bacterium diversity in activated sludge: *Nitrosococcus mobilis* and *Nitrospira*-like bacteria as dominant populations. Appl Environ Microbiol 64: 3042-3051.

2. Loy A, Schulz C, Lucker S, Schopfer-Wendels A, Stoecker K, et al. (2005) 16S rRNA gene-based oligonucleotide microarray for environmental monitoring of the betaproteobacterial order "*Rhodocyclales*". Appl Environ Microbiol 71: 1373-1386.

3. Zheng D, Alm EW, Stahl DA, Raskin L (1996) Characterization of universal small-subunit rRNA hybridization probes for quantitative molecular microbial ecology studies. Appl Environ Microbiol 62: 4504-4513.

4. Haider S, Collingro A, Walochnik J, Wagner M, Horn M (2008) *Chlamydia*-like bacteria in respiratory samples of community-acquired pneumonia patients. FEMS Microbiology Letters 281: 198-202.

5. Amann RI, Binder BJ, Olson RJ, Chisholm SW, Devereux R, et al. (1990) Combination of 16S rRNA-targeted oligonucleotide probes with flow cytometry for analyzing mixed microbial populations. Appl Environ Microbiol 56: 1919-1925.

6. Daims H, Bruhl A, Amann R, Schleifer KH, Wagner M (1999) The domain-specific probe EUB338 is insufficient for the detection of all *Bacteria*: development and evaluation of a more comprehensive probe set. Syst Appl Microbiol 22: 434-444.

7. Amann R, Snaidr J, Wagner M, Ludwig W, Schleifer K (1996) In situ visualization of high genetic diversity in a natural microbial community. J Bacteriol 178: 3496-3500.

8. Wallner G, Amann R, Beisker W (1993) Optimizing fluorescent in situ hybridization with rRNA-targeted oligonucleotide probes for flow cytometric identification of microorganisms. Cytometry 14: 136-143.
